# Actigraphy Analysis of Sleep Associates with Salivary IL-6 Concentration in Institutionalized Older Individuals

**DOI:** 10.3390/diseases11030093

**Published:** 2023-07-04

**Authors:** Vanessa Ibáñez-del Valle, Mayra Alejandra Mafla-España, Josep Silva, Omar Cauli

**Affiliations:** 1 Department of Nursing, Faculty of Nursing and Podiatry, University of Valencia, c/de Méndez y Pelayo, 19, 46010 Valencia, Spain; maria.v.ibanez@uv.es (V.I.-d.V.); maymaes@alumni.uv.es (M.A.M.-E.); 2 Frailty and Cognitive Impairment Organized Group (FROG), University of Valencia, 46010 Valencia, Spain; 3 Chair of Active Ageing, University of Valencia, 46010 Valencia, Spain; 4 Valencian Research Institute for Artificial Intelligence, Universitat Politècnica de València, 46010 Valencia, Spain; jsilva@dsic.upv.es

**Keywords:** inflammatory markers, insomnia, dementia, psychotropic drugs, polypharmacy, sleep

## Abstract

Sleep disorders are common in older individuals and are most prevalent in those who are institutionalized. Sleep complaints are often comorbid with medical and neuro-psychiatric illness and associated with polypharmacy. Various studies show an association between sleep disorders and altered levels of inflammatory cytokines, especially IL-6. In this study, an objective sleep analysis was performed using actigraphy, and IL-6 measurements in saliva in 61 older people residing in long-term nursing homes (72.1% women). Almost half (49.2%) of the people had no or mild cognitive impairment, and the rest suffered from moderate to severe dementia, mainly due to Alzheimer’s disease (25 out of 31 individuals). A significant correlation was found between salivary IL6 and sleep parameters; e.g., less salivary IL-6 had significantly (*p* < 0.05) worse sleep efficiency and more night awakenings. In turn, actigraphy detected alterations in people with dementia in average sleep time, daily bedtime, and average daily time out of bed. There was no significant correlation between these sleep patterns and the total number of psychotropic drugs. No significant differences were found in salivary IL-6 between individuals with or without dementia. These results should be considered in future research with institutionalized people to detect sleep disturbances and to establish interventions aimed to improve sleep quality.

## 1. Introduction

Sleep disturbances are common in older adults, and little is known about sleep patterns of cognitively intact older adults and its relationship to subsequent cognitive impairment [1]. Sleep is essential to the brain as it supports learning and memory, regulates synaptic plasticity, and enhances waste clearance from the brain [2]. Conversely, disturbed sleep may harm the brain through increased neuro-inflammation [3], and sleep disturbances have been associated with incident dementia [4]. Most recent studies of the brain’s glymphatic system suggest that sleep may promote the elimination of beta-amyloid peptide, which affects the pathophysiology of Alzheimer’s disease [5].

It has been estimated that up to 50% of elderly people report problems related to sleep onset (difficulty falling asleep) and sleep development, such as frequent awakening episodes [6]. Sleep disturbances are common in the elderly [7] and institutionalization in nursing care homes may contribute to increasing the risk of sleep disorders in this population [8]. Sleep disruption and fatigue have been found to predict cognitive impairment [5] or worsen it [9]. In addition, insomnia may be a risk factor for accelerated cognitive decline [10]. Insufficient hours of sleep due to insomnia or other disorders, such as sleep apnea have also been shown to be linked to an increased risk of Alzheimer’s disease, the most prevalent type of dementia [10]. Monitoring sleep in institutionalized older individuals is especially necessary for all these reasons. However, few studies have focused on this population.

Neurocognitive and sleep disorders are frequently comorbid and underdiagnosed [5]. In addition, medical and psychiatric illnesses that are common in older adults often require long-term drug therapy. Older individuals often take multiple medications, with one or more causing disrupted sleep [11]. Neuropsychiatric side-effects of drugs are not rare events among the elderly [12]. Cognitive behavioral therapy should always be the first-line treatment [13], but, when pharmacological intervention is necessary for older people with sleep disorders, careful and conservative use of drugs with sedative or hypnotic effects for the shortest practical period and with the lowest effective doses is recommended [11].

In healthcare practice, nurses apply the scientific method to provide care for the patient, family, and community in a structured, homogeneous, logical, and systematic way. One of the stages in this procedure is the assessment of vital processes or health problems that can be treated by nursing professionals, such as the patient’s sleep rest. The incorporation of objective methods to assess sleep in patients with dementia in nursing care plans is of great interest. The information provided by these methods is the basis for establishing nursing interventions for sleep hygiene. For people with moderate to severe cognitive impairment, this assessment is difficult because they have difficulty describing and interpreting their sleep quality, and they do not verbalize their sleep disturbances in interviews or report the consequences for their daily life. One of the most useful objective methods for measuring sleep in people with dementia is wrist actigraphy, which has a sensitivity of over 90% [14] and allows sleep to be assessed in the patient’s usual environment without having to subject the patient to hospital admission, which would foster stress and disorientation.

Studies of sleep quality in institutionalized elderly people using objective methods, such as actigraphy, have been limited to date. Hoyos et al. [15] conducted a case–control study to assess circadian rhythms and their association with sleep measured using actigraphy in elderly patients diagnosed with depression and patients without depression. The sleep–wake rhythms measured with actigraphy of elderly people with intellectual disabilities have also been compared with those of healthy people, with the finding that although elderly people with disabilities sleep longer, they have more fragmented and less stable sleep–wake rhythms than healthy elderly people [16]. Hoekert et al. [17] compared the use of a questionnaire (Circadian Sleep Inventory for Normal and Pathologic States) and actigraphy to quantify circadian and sleep disturbances in 78 institutionalized demented elderly people. The findings suggested a reasonable cross-validity of actigraphy and the questionnaire. Despite multiple publications on the use of actigraphy in health sciences, few studies to measure sleep using actigraphy have been conducted with institutionalized elderly people with or without dementia.

Sleep disturbance is associated with inflammatory disease risk and all-cause mortality [18]. The fact that some cytokines, such as IL-6, show a circadian pattern, with the highest concentrations at night, suggests a potential role for these molecules in the physiological regulation of sleep [19]. Various studies show an association between sleep disorders and altered levels of inflammatory cytokines measured in saliva, and the literature shows a relationship between these salivary biomarkers and sleep quality, especially in the case of IL-6, in both healthy subjects and several pathologies associated with sleep disorders [20]. Although IL-6 has been shown to be a key mediator of inflammation whose secretion can show daily fluctuations [21], there are differences in the circadian rhythm of blood and saliva IL-6, as shown in the studies by Bauer et al. [19] and Kanikowska et al. [22]. Bauer et al. [19] presented a circadian pattern of serum IL-6 concentration. Serum interleukin-6 (IL-6) concentrations over a 48 h period showed a periodicity with low values during the day and peaks in the last two-thirds of the night. Nevertheless, in the study by Kanikowska et al. [22], salivary IL-6 concentration did not display daily rhythmicity, and its concentrations did not differ significantly between the seasons (summer and winter). This difference in the circadian rhythm of blood and saliva IL-6 should be considered in biomedical studies, which are increasingly using the diagnostic potential of saliva in research.

The measurement of salivary inflammatory molecules as biomarkers of sleep alterations is an easy-to-apply and non-invasive method that can be very useful as an objective method of sleep assessment in people with cognitive impairment. However, the current scientific literature related to the measurement of IL-6 in saliva to objectively assess sleep is scarce, and only nine scientific articles that deal with this topic have been found [20]. Further studies are therefore required. This is the first study that has studied the relationship between salivary IL-6 and sleep in institutionalized elderly people with or without cognitive impairment.

The main objectives proposed in this research are as follows:

(1) To evaluate sleep with actigraphy in institutionalized older individuals, comparing the results between individuals with or without dementia.
(2) To analyze the associations between salivary IL-6 concentration and sleep parameters.
(3) To determine the influence of psychotropic medications.

## 2. Materials and Methods

### 2.1. Study Design and Population

This research project is based on a cross-sectional study conducted between September 2018 and 2019. The study population consisted of individuals aged 60 years and older residing in long-term nursing homes in the Valencia province (Spain).

This research met the requirements of the Declaration of Helsinki at all stages. The complete study and its action protocols were approved by the Ethics Committee of the University of Valencia (approval code: H1384175284261). All participants were informed about the study and they signed a form giving explicit consent for their participation. All the participants’ data were anonymized so that each participant was assigned a code X, where X is a randomly generated number. The personal and medical data were linked to the corresponding code in such a way that it was impossible for anyone with access to the data to relate them to the participant concerned.

The inclusion criteria are institutionalization ≥ 6 months and age ≥ 60 years.

Inclusion criteria concerned outpatients with AD diagnosis, meeting the Dementia of Alzheimer Type (DAT) criteria of the DSM-5 (Diagnostic and Statistical Manual for Mental Disorders, 5th edition).

The exclusion criteria are uncontrolled psychiatric illness (schizophrenia, bipolar disorder, etc.), blindness, the presence of serious infections, diagnosed cancer, active participation in a corticosteroid treatment, or inability to speak.

### 2.2. Data Collection

The information collected for each participant is summarized in Table 1. This table includes the information collected from actigraphy, saliva/blood tests, and medical reports on diseases, prescribed drugs, etc. Specifically, it includes demographic information, current and past illnesses, medical conditions, prescription of drugs (including sleep medication), and diagnosis of psychiatric and sleep disorders. Any daily activities that could affect sleep behaviors were also recorded.

### 2.3. Objective Sleep Assessment with Actigraphy

All the participants wore a wGT3XBT^®^ model actigraphy watch with firmware version v.1.5 for a week. A specialist nurse oversaw the programming of the watches and downloading the data collected, and informed the participants about how to wear the watches. The data obtained by the watches were analyzed with the ActiLife^©^ version 6.11.5 software using the following configuration:

- Sleep analysis algorithm: Cole–Kripke [23].
- Sleep period detection algorithm: Tudor–Locke [24].
- Sampling (period): 60 s.

ActiLife^®^ processed each participant’s movement activity and produced a sleep analysis. In order to extract more information and perform more analyses, ActiLife^®^ sleep reports were further processed (i) automatically, with software that we developed specifically for that purpose; and (ii) manually. This process consisted of (1) deleting the last day of data for all participants (due to being incomplete, since the actigraphs were removed at 11:00 in the morning); (2) deleting those days on which the participant did not use the watch properly; and (3) computing the relevant derived data, such as average awakening time, sleep < 4 h, etc.

**Figure 1:**
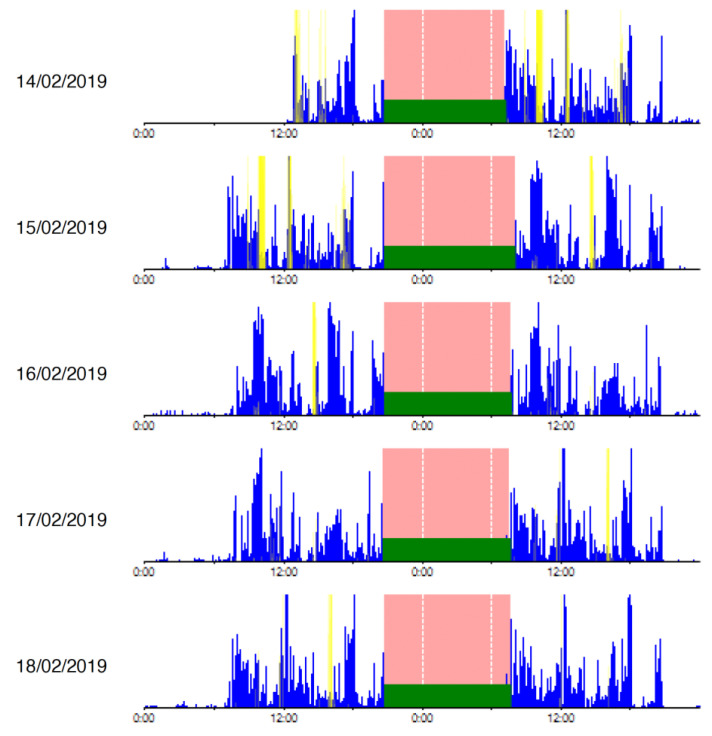
Sleep activity registered with wGT3XBT^®^ actigraph.

The average value and standard deviation of the actigraphic variables can be seen in Table 2, and an example of sleep detection carried out by ActiLife^®^ with real data from our sample of participants can be seen in Figure 1. The filled areas represent sleep periods, and they show sleep that can be considered normal and stabilized. The graph shows how sleep lasts approximately 8 h every day, with hardly any awakenings and with a stable schedule.

### 2.4. Cognitive Assessment

Lobo’s Mini-Mental State Examination (MEC) is the version of Folstein’s MMSE (Mini-Mental State Examination) adapted and validated in Spain. It is a dementia screening test. The maximum total score is 35 points, with cut-off points depending on whether the patients are geriatric or not. For geriatric patients (>65 years), presence of cognitive deterioration is considered if the score is <23 points [25].

### 2.5. Polypharmacy

For each participant, we registered the number of administered drugs that may influence sleep duration and quality: opioids, antidepressants, antipsychotics, anxiolytics, hypnotics, and antiepileptics. We also registered the total of psychotropic drugs (the sum of the previous ones) and the total drugs of any type. The number of all drugs prescribed on a daily basis was also recorded from medical data sheets.

### 2.6. Measurement of Salivary IL-6

To measure salivary IL-6 levels, saliva samples were obtained during the morning between 9:00 am and 11:00 am without fasting conditions using the Salivette^®^ system (Sarstedt, Germany). The participants were asked to refrain from smoking, eating, drinking, or oral hygiene procedures for at least 1 h prior to the sample collection. The saliva samples were processed in the lab between 10 am and 4 pm within the same day of collection. Each sample was centrifuged to remove mucins, insoluble material, and cellular debris, and the supernatant was aliquoted into Eppendorf tubes and frozen (−80 °C) until further analysis. The samples (100 uL) were brought to room temperature, and immunoassay ELISA analysis was performed using the High Sensitivity Human Elisa Kit for IL-6 (reference number Ab178013) according to the manufacturer’s instructions. Changes in color intensity and absorbance at 450 nm and 490 nm were read using the ELISA microplate reader, and a standard curve was prepared by plotting the absorbance readings of the standards against their concentration. Since several common comorbidities in older individuals are associated with an increase in inflammatory markers, including IL-6, such as arterial hypertension [26], chronic obstructive pulmonary disease [27], diabetes [28,29], dyslipidemia [30], or osteoarthritis [31,32], we evaluated whether salivary IL-6 differ based on the presence or absence of these comorbidities. Since anxiety and depression are common psychiatric disorders in this population, we also analyzed whether there was a significant association with these comorbidities, which have also been linked to altered IL-6 level [33,34].

### 2.7. Statistical Analysis

Descriptive statistical analyses were performed. The frequency distribution was calculated for the qualitative variables; for the quantitative variables, we obtained measures of central tendency (arithmetic mean), standard error of the mean (SEM), and the range of values. The normal distribution of each variable was estimated using the Kolmogorov–Smirnov test. Since none of the variables presented a normal distribution, non-parametric statistical analysis tools were used. The Mann–Whitney or Kruskall–Wallis test was used to compare the differences between the independent groups when the dependent variable is ordinal or continuous. Bivariate correlations between continuous variables were performed using Spearman’s correlation. The statistical relationship between two categorical variables was studied using Pearson’s Chi square test. All statistical tests were considered statistically significant at the *p* < 0.05 level. Analyses were carried out with the IBM Statistical Package for Social Sciences SPSS software package (version 26.0; SPSS, Inc., Chicago, IL, USA).

## 3. Results

### 3.1. Sociodemographic Data and Polypharmacy

The study included 61 people with a mean age of 82.2 ± 1.0 (SEM) (range 63–97 years), of which 72.1% were women and 27.9% were men. A total of 49.2% of the participants did not present cognitive impairment, while 18%, 23%, and 9.8% presented mild, moderate, and severe cognitive impairment, respectively. Among the 31 participants enrolled in the study with cognitive impairment, 26 had a diagnosis of Alzheimer’s disease, and the other 6 participants were classified as unknown cause of dementia. In relation to the participants with mild, moderate, and severe cognitive impairment, it was shown that 76.9% took opioids, 42.3% antidepressant medication, 15% antipsychotic medication, 30.7% anxiolytics, 61.5% % hypnotics, and 11.5% antiepileptic medication. The mean value of the comorbidities index was 6.8 ± 0.38 (SEM) (range 2–16). The prevalence of common comorbidities and the intake of drugs that potentially influence sleep quality are shown in Table 3.

### 3.2. Relationship between Sleep Parameters and Age and Gender

There were no significant correlations between mean time in bed and age (Rho = −0.10, *p* = 0.45, Spearman’s test), between mean time out of bed and age (Rho = 0.10, *p* = 0.45, Spearman’s test), between efficiency and age (Rho = −0.13, *p* = 0.33, Spearman’s test), between total sleep time and age (Rho = −0.17, *p* = 0.20, Spearman’s test), between number of awakenings and age (Rho = −0.11, *p* = 0.40, Spearman’s test), between total awakening time and age (Rho = −0.08, *p* = 0.55, Spearman’s test), between mean awakening time and age (Rho = −0.01, *p* = 0.94, Spearman’s test) or between mean time in bed when sleeping and age (Rho = −0.19, *p* = 0.14, Spearman’s test).

No significant differences were found between average daily time in bed and gender (*p* = 0.64, Mann–Whitney U test) or between average daily time out of bed and gender (*p* = 0.64, Mann–Whitney U test); on the other hand, there were significant differences between genders with regards to sleep efficiency (*p* = 0.04, Mann–Whitney U test). No significant differences were found for the other sleep parameters for gender; total sleep time (*p* = 0.19, Mann–Whitney U test), number of awakenings (*p* = 0.71, Mann–Whitney U test), total awakening time (*p* = 0.81, Mann–Whitney U test), average awakening time (*p* = 0.05, Mann–Whitney U test) and average time spent in bed when sleeping (*p* = 0.89, Mann–Whitney U test).

### 3.3. Relationship between Sleep and Polypharmacy

No significant correlations were found between the average daily time in bed for each of the psychoactive drugs: opioids (Rho = −0.01, *p* = 0.90, Spearman’s test), antidepressants (Rho = 0.07, *p* = 0.56, Spearman’s test), antipsychotics (Rho = −0.04, *p* = 0.74, Spearman’s test), anxiolytics (Rho = 0.07, *p* = 0.59, Spearman’s test), hypnotics (Rho = 0.07, *p* = 0.57, Spearman’s test) or antiepileptics (Rho = −0.10, *p* = 0.42, Spearman’s test). Likewise, there was no significant correlation between the average daily time in bed and the total number of psychotropic drugs (Rho = 0.07, *p* = 0.58, Spearman’s test) or between the average daily time in bed and the total number of drugs (Rho = −0.01, *p* = 0.93, Spearman’s test). No significant correlations were found between the average daily time out of bed and each of the psychotropic drugs: opioids (Rho = 0.01, *p* = 0.90, Spearman’s test), antidepressants (Rho = −0.7, *p* = 0.56, Spearman’s test), antipsychotics (Rho = 0.04, *p* = 0.74, Spearman’s test), anxiolytics (Rho = −0.07, *p* = 0.59, Spearman’s test), hypnotics (Rho = −0.07, *p* = 0.57, Spearman’s test) or antiepileptics (Rho = 0.10, *p*= 0.42, Spearman’s test), or between the average daily time out of bed and the total number of psychotropic drugs (Rho = −0.07, *p* = 0.58, Spearman’s test) or between the average daily time out of bed and the total number of drugs (Rho = 0.01, *p* = 0.93, Spearman’s test). No significant correlations were found between sleep efficiency and each of the psychotropic drugs: opioids (Rho = 0.13, *p* = 0.32, Spearman’s test), antidepressants (Rho = −0.03, *p* = 0.80, Spearman’s test), antipsychotics (Rho = 0.03, *p* = 0.81, Spearman’s test), anxiolytics (Rho = 0.00, *p* = 0.94, Spearman’s test), hypnotics (Rho = 0.17, *p* = 0.20, Spearman’s test) or antiepileptics (Rho = 0.17, *p* = 0.19, Spearman’s test). Likewise, there were no significant correlations between sleep efficiency and the total number of psychotropic drugs (Rho = 0.19, *p* = 0.15, Spearman’s test) or between sleep efficiency and the total number of drugs (Rho = 0.14, *p* = 0.35, Spearman’s test). There were no significant correlations between the average sleep time and each of the psychotropic drugs: opioids (Rho = −0.16, *p* = 0.22, Spearman’s test), antidepressants (Rho = −0.10, *p* = 0.45, Spearman’s test), antipsychotics (Rho = −0.02, *p* = 0.85, Spearman’s test), anxiolytics (Rho = 0.12, *p* = 0.35, Spearman’s test), hypnotics (Rho = 0.03, *p* = 0.77, Spearman’s test) or antiepileptics (Rho = −0.13, *p* = 0.32, Spearman’s test), or between the average sleep time and the total number of psychotropic drugs (Rho = −0.01, *p* = 0.90, Spearman’s test) or between the average sleep time and the total number of drugs (Rho = −0.13, *p* = 0.39, Spearman’s test). No significant correlations were found between the number of awakenings and each of the psychotropic drugs: opioids (Rho = −0.15, *p* = 0.27, Spearman’s test), antidepressants (Rho = 0.03, *p* = 0.78, Spearman’s test), antipsychotics (Rho= −0.04 *p* = 0.77, Spearman’s test), anxiolytics (Rho = 0.06, *p* = 0.62, Spearman’s test), hypnotics (Rho = −0.08, *p* = 0.52, Spearman’s test) or antiepileptics (Rho = −0.2, *p* = 0.11, Spearman’s test), or between the number of awakenings and the total number of psychotropic drugs (Rho = −0.05, *p* = 0.68, Spearman’s test) or between the number of awakenings and the total number of drugs (Rho = −0.11, *p* = 0.44, Spearman’s test). There were no significant correlations between the total awakening time and each of the psychotropic drugs: opioids (Rho = −0.08, *p* = 0.55, Spearman’s test), antidepressants (Rho = 0.03, *p* = 0.77, Spearman’s test), antipsychotics (Rho= −0.01 *p* = 0.89, Spearman’s test), anxiolytics (Rho = 0.09, *p* = 0.47, Spearman’s test), hypnotics (Rho = −0.02, *p* = 0.84, Spearman’s test) or antiepileptics (Rho = −0.15, *p* = 0.27, Spearman’s test). Likewise, no significant correlations were found between the total awakening time and the total number of psychotropic drugs (Rho = −0.03, *p* = 0.81, Spearman’s test) or between the total awakening time and the total number of drugs (Rho = −0.13, *p* = 0.39, Spearman’s test). There were no significant correlations between the average awakening time and each of the psychotropic drugs: opioids (Rho = −0.11, *p* = 0.42, Spearman’s test), antidepressants (Rho = 0.02, *p* = 0.87, Spearman’s test), antipsychotics (Rho = 0.10 *p* = 0.43, Spearman’s test), anxiolytics (Rho = 0.07, *p* = 0.61, Spearman’s test), hypnotics (Rho = 0.09, *p* = 0.51, Spearman’s test) or antiepileptics (Rho = 0.07, *p* = 0.60, Spearman’s test), or between the average awakening time and the total number of psychotropic drugs (Rho = 0.03, *p* = 0.78, Spearman’s test) or between the average awakening time and the total number of drugs (Rho = −0.03, *p* = 0.80, Spearman’s test). There were no significant correlations between the average time in bed when sleeping and each of the psychotropic drugs: opioids (Rho = −0.14, *p* = 0.29, Spearman’s test), antidepressants (Rho = −0.06, *p* = 0.63, Spearman’s test), antipsychotics (Rho = −0.01 *p* = 0.92, Spearman’s test), anxiolytics (Rho = 0.12, *p* = 0.36, Spearman’s test), hypnotics (Rho = 0.05, *p* = 0.68, Spearman’s test) or antiepileptics (Rho = −0.10, *p* = 0.43, Spearman’s test), or between the average time in bed when sleeping and the total number of psychotropic drugs (Rho = 0.03, *p* = 0.82, Spearman’s test) or between the average time in bed when sleeping and the total number of drugs (Rho = −0.11, *p* = 0.46, Spearman’s test). None of the sleep parameters was significantly related to the number of psychotropic drugs.

### 3.4. Relationship between IL-6 in Saliva with Sleep Assessment

There were no significant correlations between the average daily time in bed and the salivary concentration of IL6 (Rho = 0.13, *p* = 0.33, Spearman’s test). Likewise, there were no significant correlation between the average daily time out of bed and the salivary concentration of IL6 (Rho = −0.13, *p* = 0.33, Spearman’s test). There was a significant correlation between sleep efficiency and the salivary concentration of IL6 (Rho = 0.28, *p* = 0.03, Spearman’s test). There was no significant correlation between the average sleep time and salivary IL6 concentration (Rho = 0.05, *p* = 0.71, Spearman’s test). On the other hand, there was a significant correlation between the number of awakenings and the salivary concentration of IL6 (Rho = −0.30, *p* = 0.02, Spearman’s test). No significant correlations were found between the other sleep parameters and the salivary concentration of IL6; total awakening time (Rho = −0.21, *p* = 0.12, Spearman’s test), average awakening time (Rho = −0.05, *p* = 0.69, Spearman’s test) and average time in bed when sleeping (Rho = 0.08, *p* = 0.54, Spearman’s test). There were no significant correlations between the other sleep parameters and the salivary concentration of IL6.

### 3.5. Relationship between the Concentration of IL6 in Saliva with Age, Gender, and Comorbidities

There were no significant correlations between the concentration of IL6 in saliva and age (Rho = 0.06, *p* = 0.96) nor between women and men (*p* = 0.94). There were no significant correlations between the concentration of IL6 in saliva with comorbidities as expressed by the Charlson comorbidity index (Rho = −0.04, *p* = 0.71, Spearman correlation). Similarly, no significant differences were found between the concentration of IL6 in saliva with each of the following common comorbidities: arterial hypertension (*p* = 0.51, Mann–Whitney U test), diabetes (*p* = 0.76, Mann–Whitney U test), chronic obstructive pulmonary disease (*p* = 0.97, Mann–Whitney U test), dyslipidemia (*p* = 0.96, Mann–Whitney U test), anxiety (*p* = 0.13, Mann–Whitney U test) and depression (*p* = 0.10, Mann–Whitney U test).

### 3.6. Relationship between the Concentration of IL6 in Saliva and Cognitive Function

No significant correlations were found between the MMSE score and the concentration of IL6 in saliva (Rho = −0.13, *p* = 0.31, Spearman’s test). There were no significant differences between dementia and IL6 concentration (*p* = 0.38, Mann–Whitney U test).

**Figure 2:**
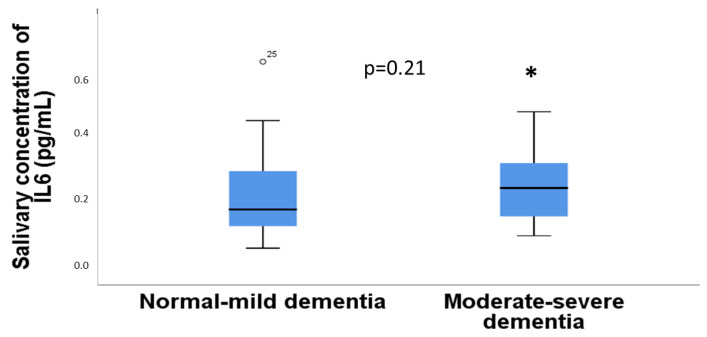
Relationship between the concentration of IL6 individuals with normal or mild dementia versus individuals with moderate to severe dementia. *p*-value (*) is indicated in the figure.

There were no significant differences between dementia in three categories and the concentration of IL6 in saliva (*p* = 0.45, Kruskal–Wallis test). Similarly, there were no significant differences between dementia in two categories and the concentration of IL6 in saliva (*p* = 0.21, Mann–Whitney U test) (Figure 2).

### 3.7. Actigraphy Sleep Parameters in Individuals with or without Dementia

There were no significant differences between dementia (cut-off < 23 points) and the average daily time in bed (*p* = 0.96, Mann–Whitney U test). Similarly, no significant differences were found between dementia and the average daily time out of bed (*p* = 0.96, Mann–Whitney U test), or between dementia and efficiency (*p* = 0.31, Mann–Whitney U test). There were no significant differences between dementia and average sleep time (*p* = 0.21, Mann–Whitney U test), between dementia and the number of awakenings (*p* = 0.40, Mann–Whitney U test), between dementia and total awakening time (*p* = 0.37, Mann–Whitney U test), between dementia and average awakening time (*p* = 0.79, Mann–Whitney U test), or between dementia and average time in bed when sleeping (*p* = 0.40, Mann–Whitney U test).

**Figure 3:**
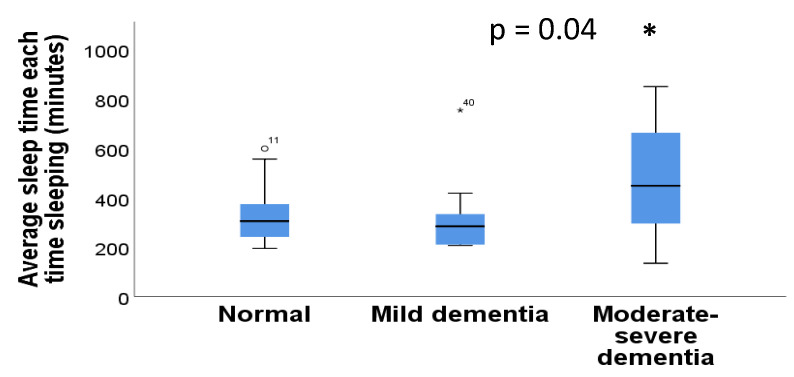
Average sleep time in individuals with or without dementia. *p*-value (*) is indicated in the figure.

The dementia variable was categorized into three variables (0 = no cognitive impairment, 1 = mild cognitive impairment, and 2 = moderate/severe cognitive impairment). No significant differences were found between dementia and average daily time in bed (*p* = 0.06, Kruskall–Wallis test). Likewise, there were no significant differences between dementia and the average daily time out of bed (*p* = 0.06, Kruskall–Wallis test), or between dementia and efficiency (*p* = 0.75, Kruskall–Wallis test). On the other hand, significant differences were found between dementia and average sleep time (*p* = 0.04, Kruskall–Wallis test) (Figure 3). No significant differences were found between dementia and the other sleep parameters: number of awakenings (*p* = 0.77, Kruskall–Wallis test), total awakening time (*p* = 0.70, Kruskall–Wallis test), average awakening time (*p* = 0.69, Kruskall–Wallis test), and average time in bed when sleeping (*p* = 0.12, Kruskall–Wallis test).

**Figure 4:**
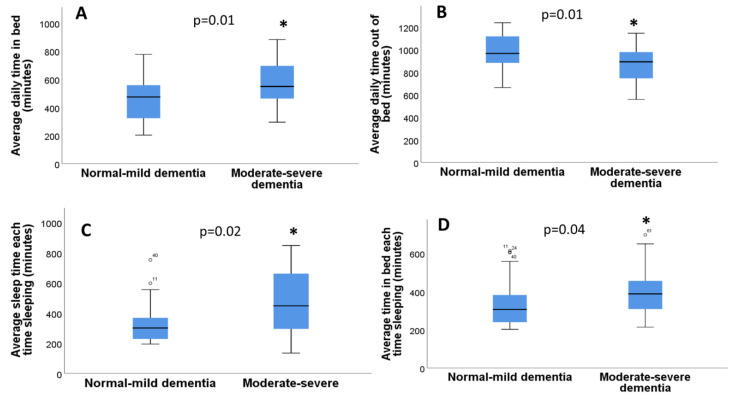
Actigraphy sleep parameters in individuals with or without dementia: (**A**) Relationship between average daily time in bed (minutes) with or without dementia; (**B**) Relationship between average daily out of time in bed (minutes) with or without dementia; (**C**) Relationship between average sleep time with dementia and without dementia; (**D**) Relationship between the average time in bed sleeping (minutes) with dementia and without dementia. *p*-values (*) are indicated in the figure.

The dementia variable was, in turn, categorized into two variables (0 = normal/mild, 1 = moderate/severe). Since the number of participants with mild cognitive impairment was small, participants without cognitive impairment were grouped with those with mild cognitive impairment and participants with moderate and severe dementia were grouped together. Significant differences were found between dementia and the average daily time in bed (*p* = 0.01, Mann–Whitney U test), and between dementia and average daily time out of bed (*p* = 0.01, Mann–Whitney U test) (Figure 4A,B). There were no significant differences between dementia and efficiency (*p* = 0.45, Mann–Whitney U test), but there were significant differences between dementia and average sleep time (*p* = 0.02, Mann–Whitney U test) (Figure 4C). No significant differences were found between dementia and number of awakenings (*p* = 0.57, Mann–Whitney U test), between dementia and total awakening time (*p* = 0.45, Mann–Whitney U test), or between dementia and average awakening time (*p* = 0.74, Mann–Whitney U test). In addition, there were significant differences between dementia and the average time in bed when sleeping (*p* = 0.04 Mann–Whitney U test) (Figure 4D).

### 3.8. Dementia and Polypharmacy

No significant differences were found between dementia and antidepressants (*p* = 0.51, Mann–Whitney U test). There were significant differences between dementia and antipsychotics (*p* = 0.05, Mann–Whitney U test). There were no significant differences between dementia and the other psychotropic drugs; anxiolytics (*p* = 0.22, Mann–Whitney U test), hypnotics (*p* = 0.56, Mann–Whitney U test), or antiepileptics (*p* = 0.36, Mann–Whitney U test). On the other hand, significant differences were found between dementia and the total number of psychotropic drugs (*p* = 0.04, Mann–Whitney U test), but there were no significant differences between dementia and the total number of drugs (*p* = 0.41, Mann–Whitney U test).

No significant differences were found between dementia in three categories (0 = no cognitive impairment, 1 = mild cognitive impairment, and 2 = moderate/severe impairment) for each of the psychoactive drugs: opioids (*p* = 0.39, Pearson’s Chi square), antidepressants (*p* = 0.73, Chi square test), antipsychotics (*p* = 0.58, Pearson’s Chi square), anxiolytics (*p* = 0.15, Pearson’s Chi square) and between dementia and hypnotics (*p* = 0.15, Pearson’s Chi square).

There were no significant differences between dementia in two categories (0 = normal/mild, and 1 = moderate/severe) and opioids (*p* = 0.79, Pearson’s Chi square), between dementia and antidepressants (*p* = 0.92, Pearson’s Chi square), between dementia and antipsychotics (*p* = 0.33, Pearson’s Chi square), between dementia and anxiolytics (*p* = 0.22, Pearson’s Chi square), between dementia and hypnotics (*p* = 0.51, Pearson’s Chi square) and between dementia and antiepileptics (*p* = 0.83, Pearson’s Chi square).

## 4. Discussion

To date, this is the first study that has studied the relationship between salivary IL-6 and sleep in institutionalized elderly people with and without cognitive impairment. The main findings of our study include a significant correlation between IL6 in saliva with some parameters of sleep objective assessment, e.g., sleep efficiency and the number of awakenings. People with less salivary IL-6 had worse sleep efficiency and more awakenings. Although some research has shown that sleep deprivation and sleep disorders increase daytime plasma IL-6 levels in adults [35], most studies relating IL-6 to sleep have measured IL-6 in blood and not in saliva. As shown in the recent review by Ibáñez del Valle et al. [20], these studies are inconclusive, and further studies are required to determine the sensitivity of salivary versus blood inflammatory markers in monitoring biological rhythms and acting as biomarkers in the detection of sleep disorders. Approximately fifty percent of the studies that analyzed IL-6 levels showed an increased elevation of IL-6 levels during the day in people with sleep disorders. In the others, either no significant changes were found, or results similar to our study were obtained, such as in a study carried out with children and adolescents, and the levels of salivary cytokines were found to be higher in people who had better sleep [36]. There are no studies that have been performed specifically in healthy elderly people with which these results can be compared, and no studies of this type been conducted in older people with dementia. As for studies that have assessed sleep and measured plasma IL-6 in older people, Zhang et al. [37] measured the plasma levels of biomarkers for predicting the risk of developing neurodegenerative diseases in healthy controls and in iRBD (idiopathic REM sleep behavior disorder) patients aged over 50 years. As in our study, IL-6 levels were lower in the people with IRBD than in the healthy controls. Although in our study IL-6 measurements were taken from saliva and not plasma, a recent study by Parkin et al. [38] performed in older individuals revealed significant serum–saliva correlations for several cytokines, including IL-6. Future sleep studies performed in institutionalized persons should test further the associations between salivary cytokine concentrations, such as IL-6, and sleep quality and analyze if the concentration could help to detect sleep disorders in those people unable to communicate properly about sleep quality. In future studies, it would also be interesting to consider the relationship between IL-6 and dementia. Some recent studies, such as the longitudinal study by Pedersen et al. [39] have shown that IL-6 is involved in dementia. In this research, four inflammatory biomarkers associated with Alzheimer’s disease were found, and among them was IL-6. However, as our study is a cross-sectional research design, we cannot establish a cause–effect relationship between IL-6 levels and the onset or progression of dementia.

Several studies have shown that sleep disorders may contribute to cognitive impairment or dementia, and their early detection could prevent this. Various factors, such as difficulty falling asleep, poor sleep quality, sleep loss, excessive daytime sleepiness, and sleep-disordered breathing, have been found to increase the biomarkers of Alzheimer’s disease in the elderly without dementia [40]. Sleep assessment of institutionalized persons should be performed routinely for this reason. Among the methods of sleep assessment in institutionalized persons, actigraphy is an objective assessment method that allows sleep to be analyzed objectively in people with and without dementia. In cases in which cognitive impairment does not allow the individual to complete questionnaires or sleep diaries to reach an accurate diagnosis, actigraphy can be very useful since it provides longitudinal sleep data over multiple nights, and it is a less expensive and a less cumbersome method for measuring sleep than polysomnography [41]. On the other side, chronic pain is a major issue affecting more than 50% of the older population and up to 80% of nursing home residents [42]. It would have been interesting to assess the influence of the participants’ level of pain, the use of all analgesic drugs that could affect sleep, and the increase in IL-6 values of the participants in our study. In the case of opioids intake, we did not observe any significant effect on IL-6 levels and sleep parameters; but obviously, larger study samples are needed to elucidate this issue in detail. Regarding the role of comorbidities relevant to the study’s outcomes, future studies should evaluate the presence of knee osteoarthritis [32] and its effects on IL-6 levels and sleep quality in individuals with or without comorbid dementia. According to the medical records, we were unable to measure whether the variables studied differed between people with and without osteoarthritis. Because many of the patients enrolled in our study did not walk much, the diagnosis of knee osteoarthritis was underdiagnosed in this population [43,44]. Another research limitation regarding the role of comorbidities was the failure to include individuals with different weight groups (for instance, only three individuals were classified as obese in the group of individuals without dementia and one in the group of dementia). Obesity is characterized by chronic low-grade inflammation with a moderate increase in circulating levels of IL-6 [45], which can contribute to a rise in IL-6 and impairs sleep quality [46,47].

Sleep complaints are often comorbid with medical and psychiatric illnesses associated with the medications used to treat those illnesses [11]. However, in our study, no significant correlations were found between the average daily time in bed and each of the psychoactive drugs. Likewise, there was no significant correlation between the average daily time in bed and the total number of drugs, or the total number of psychotropic drugs.

In our study, actigraphy allowed us to objectively assess sleep in institutionalized older individuals with dementia and different degrees of dementia (mild and moderate or severe). After comparing the measurements of people without dementia to those with some degree of dementia, actigraphy detected alterations in people with moderate and severe dementia in the following sleep parameters: average sleep time, daily time in bed, and average daily time out of bed. No significant differences were found between dementia with the rest of the sleep parameters: number of awakenings, total awakening time, average awakening time, and average time in bed when sleeping. It was found that people with some degree of dementia spend more time in bed on average. These results are similar to those of Lysen et al.’s group [48], in which a longer time in bed, also measured with actigraphy, was associated with an increased risk of dementia.

The longitudinal associations of time in bed (TIB) with dementia and cognitive decline in older adults are unclear [49]. Studies, such as Diem et al. [1] find that longer sleep latency was related to a higher probability of developing cognitive impairment, but total sleep time measured with actigraphy was not associated with the probability of cognitive development. However, in our study, the average sleep time correlated with the degree of dementia, so that people with some degree of dementia slept for more hours than people without dementia or with mild dementia. There are recent studies that agree with our results, and an association between the number of hours of sleep and the development of dementia has been reported. This is true of the study by Lui et al. [50] which suggests that the time people go to bed and the number of hours they sleep may affect their risk of developing dementia. In this study, the risk of dementia was 69% higher in those who slept more than 8 h (vs. 7–8 h) and two times higher in those who went to bed before 9 pm (vs. 10 pm or later). In this study, sleep parameters were assessed via subjective methods, such as standard questionnaires.

Cognitive impairment can worsen sleep hygiene. Sleep disorders are common in dementia patients, and dementia medications affect sleep [5]. Nursing home residents stay awake in bed for prolonged periods [51]. Some of the signs that are related to sleep disorder in people with dementia may include a loss of physical function and reduced participation in activities during daytime [52]. Similar results have been seen in our stud, wherein people with moderate/severe dementia spend less daily time out of bed. This constitutes inadequate sleep hygiene that needs to be addressed.

Alterations in sleep may be a fundamental substrate or major relative risk for some neurodegenerative causes of dementia, and some of the involved conditions are treatable [5]. Sleep hygiene refers to several sleep habits that can be performed to enhance sleep. These habits include increasing daytime activity and physical exercise [53,54]. Individualized social activity intervention (ISAI), which includes increased social activities, such as participation in an hour of play, has shown improvements in nighttime sleep in people with dementia [55]. Sleep hygiene measures are first-line actions to improve sleep habits and should be considered in future studies on sleep in people with dementia.

## 5. Conclusions

Dementia exacerbates sleep/wake pattern disturbances [55], and thus, sleep assessment is the cornerstone of identifying and developing any healthcare plan for these individuals. However, people with dementia are often unable to communicate their sleep patterns, and assessment is very difficult and unreliable with interviews. In these cases, sleep analysis with actigraphy is a very useful tool to measure sleep objectively. Our pilot study has evaluated sleep in people institutionalized in nursing homes with different levels of cognitive impairment. This research has the added value of having objectively assessing sleep using two methods: actigraphy and a salivary inflammatory biomarker, IL-6, which is associated with sleep efficiency and the number of awakenings. The main drawback of the actigraphy assessment was the lack of cooperation of some of the patients with dementia (as wearing a watch for seven days is not an easy task for these people). In this context, we recommend the use of actigraphy to analyze sleep and to evaluate the effects of interventions aimed to improve sleep quality in these individuals. Analysis of a panel of other cytokines in saliva and probably in the blood may help to shed new light on the pathophysiology of poor sleep quality in these individuals.

## Figures and Tables

**Table 1:** Information collected for each participant (variables analyzed).

Demographics	Actigraphy	Drugs
Gender Age Marital status	Time in bed Time out of bed Efficiency Total time in bed Total sleep time Waking after sleep onset Number of awakenings Avg. awakening time	Antipsychotics Antidepressants Hypnotics Anxiolytics Antiepileptics Opioids Analgesics
**Comorbidities**	**Cognitive assessment**	**Saliva Test**
Report	Lobo’s Mini-Mental State Examination (MEC)	IL-6

**Table 2:** Sleep variables measured with actigraphy.

Sleep Variable	Mean and Standard Error of the Mean (Min-Max Range)
Average Daily Time In Bed	497 ± 22.3 (202–882 min)
Average Daily Time Out of Bed	942 ± 22.3 (558–1238 min)
Efficiency	95.4 ± 0.33 (88–100)
Average Sleep Time Each Time Sleeping (includes naps and split sleep)	373.9 ± 23.3 (135.848 min)
Number of Awakenings	5.6 ± 0.44 (1–15 times)
Total Awakening Time	14.7 ± 1.19 (1–51 times)
Average Awakening Time	2.49 ± 0.12 (0.29–5.57)
Average Time in Bed Each Time Sleeping (includes naps and split sleep)	360 ± 17.3 (202–697 min)

**Table 3:** Comorbidities and drugs intake.

Variables		Frequency % (Categorical Variables) or Mean and Standard Error of the Mean (Range Min-Max) (Discrete Variables)	
HTA	Yes	60.7%	
	No	39.3%	
Chronic obstructive pulmonary disease	Yes	14.8%	
	No	85.2%	
Diabetes	Yes	49.2%	
	No	50.8%	
Dyslipidemia	Yes	41%	
	No	59%	
Cognitive impairment:	Normal	49.2%	
	Mild	18%	
	Moderate	23%	
	Severe	9.8%	
Depression	Yes	21.3%	
	No	78.7%	
Anxiety	Yes	8.2%	
	No	91.8%	
Charlson comorbidity index		6.87 ± 0.38 (2–16)	
Polypharmacy	Opioids	Yes	77%
		No	23%
	Antidepressants	Yes	45.9%
		No	54.1%
	Antipsychotics	Yes	27.9%
		No	72.1%
	Anxiolytics	Yes	41%
		No	59%
	Hypnotics	Yes	54.1%
		No	45.9%
	Antiepileptic	Yes	16.4%
		No	83.6%
Number of daily psychotropic drugs		3.07 ± 0.23 (0–8)	
Total number of drugs prescribed daily		11.1 ± 0.65 (3–24)	

## Data Availability

The raw data supporting the results of this paper will be made accessible upon request by the corresponding author based on scientific purposes.
